# Dual-Use Research as a Wicked Problem

**DOI:** 10.3389/fpubh.2014.00113

**Published:** 2014-08-04

**Authors:** Gregory D. Koblentz

**Affiliations:** ^1^School of Policy, Government, and International Affairs, George Mason University, Fairfax, VA, USA

**Keywords:** dual-use research of concern, biosecurity, wicked problems, H5N1, bioterrorism

The challenge of dual-use research in the life sciences emerged vividly in 2011 as scientists and policy-makers debated what to do about article manuscripts that described how to modify the H5N1 avian influenza virus so that it could spread between mammals (1, 2). Since H5N1 emerged in Southeast Asia in 2003, it has sickened 667 people and caused 393 human deaths, as well as the deaths of millions of domestic and wild birds (3). The virus has not, however, demonstrated the ability to engage in sustained human-to-human transmission. If a new strain of H5N1 emerged with that capability, and it retained a high level of virulence, it could cause a global pandemic. The experiments by Yoshihiro Kawaoka from the University of Wisconsin-Madison and Ron Fouchier from Erasmus Medical Center in the Netherlands not only demonstrated that mammalian transmission of the virus was possible but also provided information on how to construct such a virus.

These H5N1 experiments are only the latest demonstration of the dual-use dilemma at the heart of the biotechnology revolution: research conducted for peaceful purposes has the potential to be misused for malicious purposes. The H5N1 controversy highlighted the widely divergent views on the benefits and risks of dual-use research held by different stakeholders, including scientists, publishers, biosecurity experts, the national security community, and public health officials. On the one side, proponents of the research focused on the public health benefits of knowing that H5N1 can be transmitted between mammals and which specific mutations can confer this ability on the virus. Opponents of the research highlighted the risks of a laboratory accident and the potential for a nefarious actor such as a terrorist group or rogue scientist to replicate the research and deliberately release the virus.

The concept of wicked problems provides a new lens for understanding the public policy challenges posed by dual-use research. This concept was first introduced in the 1970s to describe the challenges posed by poverty, urban development, and other social issues (4). Wicked in this context does not mean evil or cool, but instead refers to the intrinsic properties of an issue that make it resistant to long-lasting solutions.

Wicked problems are characterized by multiple, overlapping subsets of problems and high levels of social complexity driven by the number and diversity of players involved in problem-solving. The parties who have a vested interest in how (or whether) the problem is solved are likely to come from different organizations and disciplines with different values and objectives so they will define the problem and acceptable solutions differently. The complex interactions between interconnected issues and the diversity of stakeholder preferences impede the wide acceptance of a definitive statement of the problem. As a result, wicked problems tend to defy traditional linear methods of problem-solving, which rely on a clear specification of the problem to drive the data collection and analysis process. Furthermore, the environment in which stakeholders are trying to solve a wicked problem is dynamic. The constraints on the solution, such as availability of resources and political ramifications, change over time, and stakeholders enter and exit the problem-solving process, change their preferences, or otherwise change the rules by which they address the problem. Since there is no definitive statement of the problem, there can be no definitive solution. As a result, the problem-solving process ends only when stakeholders run out of time, money, or energy, not when the perfect solution emerges. In addition, solutions to wicked problems are at high risk of having unanticipated effects. Wicked problems are never permanently solved since solutions have implications for other policy domains, which can generate feedback loops or have unintended consequences. The potential for this type of ripple effect increases the scope of stakeholders affected directly or indirectly by policy-making and creates the need for a wider array of information from a broader range of sources to identify the universe of potential solutions and their costs and benefits. In sum, wicked problems are “ambiguous, fluid, complex, political, and frustrating as hell.” [(5): p. 2].

Based on these characteristics, dual-use research has all of the signs of being a wicked problem. As two congressional researchers (6) wrote about the H5N1 controversy:

The current issues under debate cut across traditional policy areas, involving simultaneous consideration of security, scientific, health, export, and international policy. Because of the complexity of these issues, analysis according to one set of policy priorities may adversely affect other policy priorities (p. 24).

While wicked problems defy easy and long-lasting solutions, there are several strategies that can be used to manage them. The choice of strategy is dictated by two factors: how concentrated or dispersed power is among stakeholders and how strongly stakeholders struggle for power amongst themselves (5). Based on these criteria, four coping strategies for wicked problems can be identified: authoritative, hegemonic, competitive, and collaborative (see Table 1).

**Table 1:** **Coping strategies for wicked problems**.

	Power is concentrated	Power is dispersed
Power is contested	Hegemonic	Competitive
Power is not contested	Authoritative	Collaborative

Stakeholders following an authoritative strategy cede the authority to define and solve the problem to a small group of experts. Reducing the number of stakeholders involved in decision-making simplifies and speeds up the process. The use of experts also increases the perceived objectivity, and therefore, legitimacy of the outcome. A drawback to this strategy is that even experts can be wrong or have too narrow of a view (5).

The George W. Bush Administration initially employed an authoritative strategy to address dual-use research. In 2002, the National Academy of Sciences was commissioned to provide recommendations for how to balance the costs and benefits posed by dual-use research. As a result of this study, the Bush administration created the National Science Advisory Board for Biosecurity (NSABB) to advise the government on dual-use research oversight. Between 2005 and 2012, NSABB was at the forefront of dual-use research oversight, education, and outreach activities.

When one party is so powerful that it is able to impose its preferred problem definition and solution on other stakeholders, it can employ a hegemonic strategy. While other stakeholders may disagree with the way a problem is defined or solved, the hegemonic strategy simply excludes them from the decision-making process. The main advantage of this strategy is its speed and simplicity: problem-solving by decree. The major disadvantage of this approach is that it is more likely to try to “tame” a wicked problem than actually solve it.

In late 2004, spurred by fears that recent breakthroughs in gene synthesis technology could be used to create dangerous pathogens from scratch, the U.S. Congress made it illegal to synthesize the variola virus, which was defined as “a virus that can cause human smallpox or any derivative of the variola major virus that contains more than 85% of the gene sequence” of variola (7). Scientists objected that the seemingly precise language of the new law could potentially cripple research on smallpox vaccine and other orthopoxviruses since all of these viruses are closely related. This hegemonic strategy was a heavy-handed attempt to “tame” the problem posed by the growing sophistication of synthetic biology by simply outlawing a specific use of the technology.

When power is dispersed and contested, stakeholders view problem-solving as a zero-sum game. Stakeholders pursue a competitive strategy to consolidate their own power in order to define the problem in their preferred way and impose their preferred solution. This strategy can result in more innovative policies due to the struggle by stakeholders to persuade others of their preferred definition and solution. Another advantage of this strategy is that it impedes the centralization of power and creates opportunities for reform when the balance of power among stakeholders shifts. A disadvantage of this strategy is that it is likely to end in stalemate as different stakeholders maneuver to implement their preferred approach and block others from doing likewise (5). The competitive strategy is the default setting for resolving wicked problems in the American political system.

The 2011 controversy over the H5N1 experiments marked a shift from an authoritative to competitive strategy for dealing with dual-use research. The debate over these experiments quickly moved beyond the NSABB and the small community of biosecurity specialists to include the World Health Organization, politicians, scientific publishers, and the scientific community, especially influenza researchers. In an explicit acceptance that the scientific authority of the influenza community was no longer sufficient to shield it from oversight, Dr. Anthony Fauci, director of the National Institutes for Allergies and Infectious Disease (NIAID), told international influenza experts, “The flu scientific community can no longer be the only players in the discussion of whether the experiments should be done.” (8). At the same time, the NSABB’s charter was revised to remove its authority to review dual-use experiments and it has not met since late 2012 (9).

Collaborative strategies are best suited for situations where power is dispersed but not contested. Under these conditions, stakeholders can move beyond the zero-sum mentality and work together for “win–win” outcomes. This strategy seeks to alter the structure of payoffs to encourage cooperation through repeated iterations to build up trust or create linkages between unrelated issues to expand the potential gains achievable through cooperation. Collaboration can enable stakeholders to achieve results they would not have been capable of reaching on their own and to do so more efficiently. Increasing the number of stakeholders and seeking solutions that are acceptable to as many parties as possible increases transaction costs and delays decision-making. An additional hurdle to collaboration is that each stakeholder brings practice-based “local knowledge” to the table, which is hard to share and difficult for other stakeholders with different identities to internalize (10). Despite these disadvantages, a collaborative strategy has the potential to yield longer lasting policies that are more widely accepted by the relevant stakeholders (5).

Unfortunately, people often have to fail into collaboration. According to Roberts (5),

People have to learn what does not work before they are willing to absorb what they perceive to be the extra ‘costs’ associated with collaboration. This learning is especially important for people who come from cultures that place a high premium on taking charge, making decisions, being competitive, and using authorities and experts to settle whatever disputes arise (p. 12).

Although the authoritative strategy for addressing the wicked problem posed by dual-use research has now run its course, it is unclear what will replace it. The scientific community views the competitive and hegemonic strategies with a mixture of fear and contempt: contempt for the push-and-pull of politics that privileges sound bites over the complexities of science and fear of draconian solutions imposed by scientifically ignorant politicians and bureaucrats.

Successful collaboration on dual-use research is more likely to emerge if stakeholders engage in intensive dialog as a means of building a shared understanding about the problem and a shared commitment to solving it. Dialog is not an instrument for decision-making or a negotiating tactic to lead to agreement, but an integral part of the process of creating a shared vision among a diverse group of stakeholders. Getting the right answer is not as important as having stakeholders accept whatever solution emerges (11).

Collaboration can also be facilitated by the emergence of a “collaborative capacity builder” whose role is to ensure the integration of knowledge among stakeholders as part of a long-term strategy to foster a collaborative environment for continuously addressing the dilemmas posed by dual-use research. An individual or organization is empowered to play the role of collaborative capacity builder due to its legal authority, expertise valued by other stakeholders, reputation as an honest broker, or some combination of these values (10).

Recognizing that a problem is wicked is the first step to coping with the problem. Viewing dual-use research as a wicked problem highlights the need for stakeholders to engage in dialog with one another and to adopt collaborative strategies for managing risks in this area. Admitting that the experts do not have all of the answers and giving up the zero-sum view that dominates policy-making in a pluralistic society will be difficult, but the potential benefits of seeking collaborative solutions is well worth the discomfort caused by this mode of problem-solving.

## Conflict of Interest Statement

The author declares that the research was conducted in the absence of any commercial or financial relationships that could be construed as a potential conflict of interest.
